# Investigation of Cutting Force in Longitudinal-Torsional Ultrasonic-Assisted Milling of Ti-6Al-4V

**DOI:** 10.3390/ma12121955

**Published:** 2019-06-17

**Authors:** Ying Niu, Feng Jiao, Bo Zhao, Guofu Gao

**Affiliations:** School of Mechanical and Power Engineering, Henan Polytechnic University, Jiaozuo 454000, China; niuying@hpu.edu.cn (Y.N.); gaogf@hpu.edu.cn (G.G.)

**Keywords:** longitudinal-torsional ultrasonic vibration, milling, Ti-6Al-4V, cutting force, theoretical prediction model

## Abstract

In this study, we propose a longitudinal-torsion ultrasonic-assisted milling (LTUM) machining method for difficult-to-cut materials—such as titanium alloy—in order to realize anti-fatigue manufacturing. In addition, a theoretical prediction model of cutting force is established. To achieve this, we used the cutting edge trajectory of LTUM to reveal the difference in trajectory between LTUM and traditional milling (TM). Then, an undeformed chip thickness (UCT) model of LTUM was constructed. From this, the cutting force model was able to be established. A series of experiments were subsequently carried out to verify this LTUM cutting force model. Based on the established model, the influence of several parameters on cutting force was analyzed. The results showed that the established theoretical model of cutting force was in agreement with the experimental results, and that, compared to TM, the cutting force was lower in LTUM. Specifically, the cutting force in the feed direction, Fx, decreased by 24.8%, while the cutting force in the width of cut direction Fy, decreased by 29.9%.

## 1. Introduction

Titanium alloy has a number of excellent properties, such as corrosion resistance, high strength and good heat resistance, and is widely used in medical, aerospace and other fields [1]. It is considered a typical difficult-to-cut material due to its chemical, physical and mechanical properties; for instance, the cutting temperature and cutting force is high, the friction force is large, and tool wear is serious [2,3]. Currently, a number of difficulties and challenges still exist in the traditional processing technologies (turning, grinding and milling, etc.) for such difficult-to-cut materials, particularly while maintaining high quality, high efficiency and low cost [4,5].

Ultrasonic-assisted machining technology is a non-traditional machining technology. It applies vibrations of an ultrasonic frequency to the tool or workpiece along a certain direction. Compared with traditional cutting technologies, such as turning, grinding and milling, ultrasonic machining technology has obvious technological advantages, including a small cutting force, low cutting temperature, high surface quality and high machining precision [6,7,8].

Ultrasonic vibration-assisted milling technology started relatively recently, with its literature mainly focusing on the early 21st century. At present, research on ultrasonic vibration-assisted milling is in the developing stages. In 2006, Chern and Chang [9] studied the machinability of ultrasonic vibration-assisted micro-milling of aluminum alloy, and found that vibration milling meaningfully improved the surface quality. This was the first time that ultrasonic vibration was introduced into milling. Shen et al. [10] carried out a detailed study on ultrasonic vibration-assisted milling of aluminum alloy. They found that ultrasonic vibration-assisted milling transformed the continuous cutting process into discontinuous differential cutting. In addition, the cutting process produced a cutting force similar to that of the pulse type, effectively reducing the average cutting force. Zarchi et al. [11] studied ultrasonic vibration-assisted milling of AISI 420 stainless steel and found that for a small feed rate, it reduced the cutting force in up milling, while during a larger feed rate, it reduced the cutting force in down milling. Zhao et al. [12] investigated the cutting force differences between rotary ultrasonic machining and conventional diamond side grinding, drilling and face grinding on K9 glass, and found that rotary ultrasonic machining reduced the cutting force to a certain extent.

However, in one-dimensional ultrasonic-assisted machining, with the necessary high-frequency vibration of the tool, the tool flank generates extrusion friction with the machined surface. This is seriously harmful to the life of the tool and the machined surface quality [13,14], and limits further application of ultrasonic vibration cutting technology. On the contrary, in longitudinal-torsional ultrasonic vibration-assisted milling (LTUM), due to the cutting edge moving in three-dimensional space, extrusion friction between the tool flank and the machined surface is effectively avoided. In addition, the shear angle is increased and the average cutting force and cutting temperature in processing are (further) reduced [15,16], which is important in anti-fatigue manufacturing of high performance components.

An accurate prediction of cutting force is conducive to the evaluation of machine tool power, main bearing pressure, parts deformation, control of processing quality, and so on. At present, the most commonly used methods for predicting milling force are as follows: Micro-element model, finite element model, multiple regression analysis model and artificial neural network prediction model.

Liu et al. [17] conducted verified experiments and presented a mechanistic model for cutting force in rotary ultrasonic machining of brittle materials, and found that the model predicted cutting force with high precision. Zhang et al. [18] developed a mechanistic model to predict the cutting force in rotary ultrasonic drilling of brittle materials, showing that the model could be successfully applied to evaluate cutting force. Xiao et al. [19] developed a theoretical model of cutting force both in the axial and feed directions in rotary ultrasonic machining. Yuan et al. [20], Cong et al. [21], and Wang et al. [22] each presented a predictive model for cutting force, wherein their calculated results were subsequently verified by experimentation.

In the literature, there have been numerous valuable researches into cutting force; however, in terms of the outstanding advantages of LTUM (in particular, the tool as a vibration carrier), few of these investigations have been in relation to milling of difficult-to-cut materials, such as titanium alloys. Therefore, in the present work, in order to meet the compressive stress and anti-fatigue manufacturing requirements of titanium alloy Ti-6Al-4V, an LTUM machining method (with the tool as a vibration carrier) was developed. Models of the cutting edge trajectory and undeformed chip thickness (UCT) were also constructed. In addition, a cutting force model of LTUM was established and then verified through a series of experiments. Based on this established model, the influence of several parameters on cutting force was analyzed.

## 2. Materials and Methods

### 2.1. Trajectory Model of Cutting Edge in LTUM

**Figure 1:**
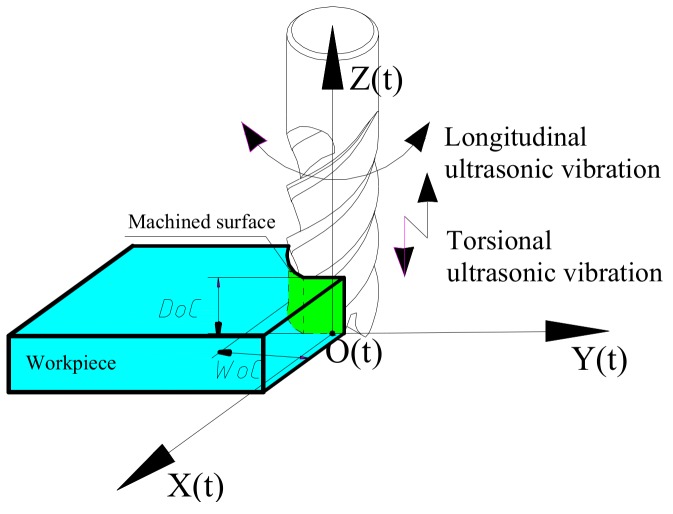
Longitudinal-torsional ultrasonic vibration-assisted milling.

The principles of LTUM are shown in Figure 1. The cutting edge trajectory employed in LTUM makes it very different to traditional milling (TM), as the trajectory used has a direct impact on the cutting force, as well as other processing procedures and results.

For TM, the cutting edge trajectory equation (Equation (1)) is as follows:

(1) $$\left\{\begin{array}{l} x\left(t\right){=v}_{f}{t+R}_{t}\sin\left(2\pi \mathrm{nt}/60\right)\\ y\left(t\right){=R}_{t}\cos\left(2\pi \mathrm{nt}/60\right)\\ z\left(t\right)=0 \end{array}\right.$$

where v_*f*_ is feed rate, R_t_ is tool radius, and n is spindle speed.

From this, an equation (Equation (2)) concerning the cutting edge trajectory for LTUM may be established:

(2) $$\left\{\begin{array}{l} x\left(t\right){=v}_{f}\cdot {t+R}_{t}\cdot \sin\left(\omega_{n-t}\right)\\ y\left(t\right){=R}_{t}\cdot \cos\left(\omega_{n-t}\right)\\ z\left(t\right){=A}_{l}\sin\left(2\pi ft\right) \end{array}\right.$$

where *f* is ultrasonic frequency, A_l_ is longitudinal amplitude in LTUM, and ω_n-t_ is the actual rotation angle of the tool, obtained from Equation (3).

(3) $$\omega_{n-t}=2\pi \left(\mathrm{nt}/60\right){+\omega }_{l-t}$$

where, ω_l-t_ is torsional angle, obtained from Equation (4).

(4) $$\omega_{l-t}{=A}_{t}\cdot \cos(2\pi f{t+\varphi }_{l-t})$$

where, A_t_ is torsional amplitude and φ_l-t_ is the phase difference for the longitudinal-torsional vibration.

**Figure 2:**
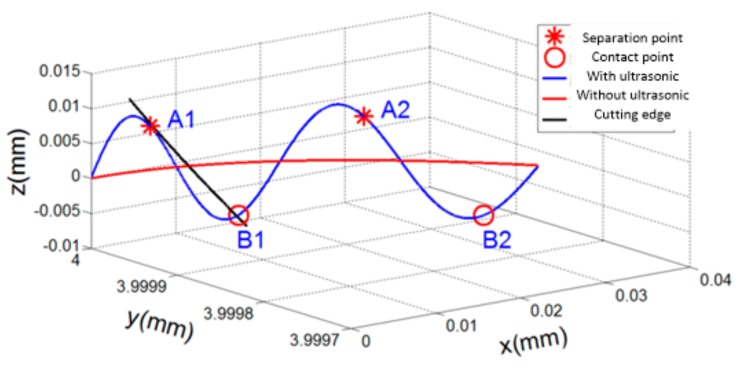
Cutting edge trajectories of longitudinal-torsion ultrasonic-assisted milling (LTUM) and traditional milling (TM).

Based on Equations (1) and (2), the trajectories of the cutting edges in LTUM and TM are illustrated in Figure 2.

In Figure 2, A1, A2,…, An points represent the starting separation positions between the tool and workpiece, while B1, B2,…, Bn points represent the starting contact points, called the feature points. It can be seen that the cutting edge moves along the blue line in LTUM, and that the tool–workpiece undergoes periodic changes of separation-contact-separation with ultrasonic vibration. This caused a large difference from the cutting edge trajectory of TM (red line).

**Figure 3:**
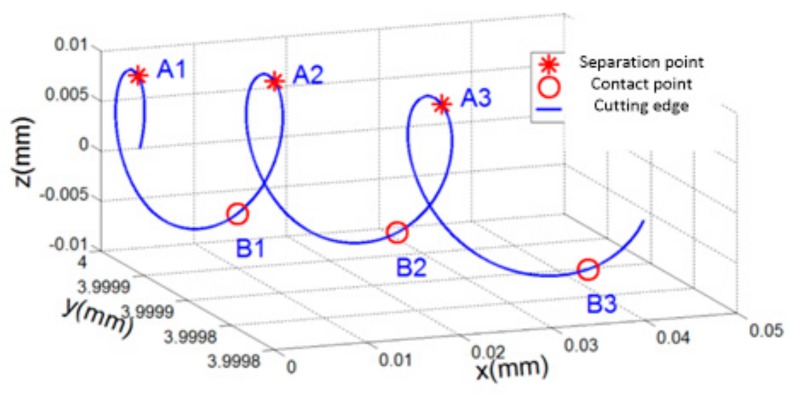
Cutting edge trajectory characteristics in LTUM.

According to the solution model of feature points, the separation and contact points of LTUM were able to be calculated, as shown in Figure 3. In the figure, A1, A2, and A3 are the separation points between the tool and workpiece, and B1, B2 and B3 are the contact points. In the process, the cutting edge is separated from the workpiece from point A1 to point B1, and contacting the workpiece from point B1 to point A2. 

Thus, we concluded that for LTUM, the tool and workpiece exhibit periodic separation, while at the same time perform a cutting edge motion in 3D space. This significantly avoids the extrusion friction between the tool flank and machined surface, thereby reducing the cutting force and improving the quality of the machined surface.

### 2.2. Undeformed Chip Thickness Model of LTUM

Undeformed chip thickness (UCT) greatly influences both the cutting force and machining results; however, compared with TM, UCT is more complicated in LTUM. As such, it is helpful to establish a UCT model of LTUM to reveal the mechanism of the cutting force. 

**Figure 4:**
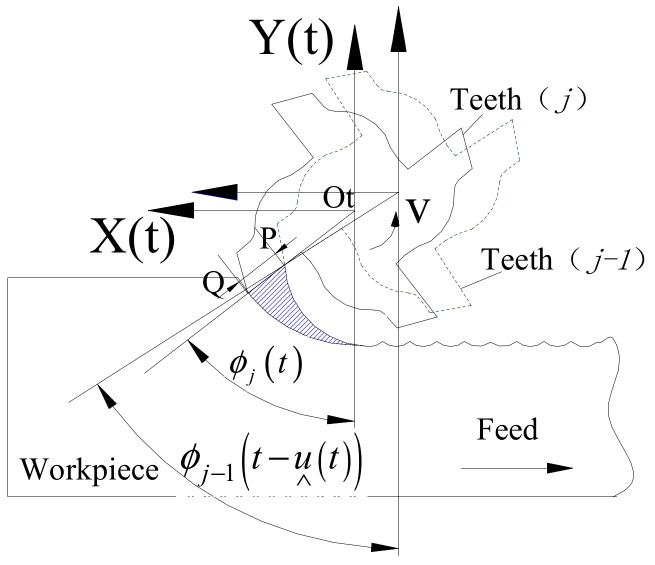
Model of undeformed chip thickness.

As shown in Figure 1, a tool coordinate system was established, wherein the feed direction was represented by the *x* axis, the width of cut direction was represented by the *y* axis, the spindle axis was represented by the *z* axis, and the origin was set on the tool rotation center. From this, a UCT model could be established, as shown in Figure 4.

At any time, t, the position of the j-th teeth is given by the solid line in Figure 4. The angular displacement of the tool teeth and the Y_t_ axis is expressed in Equation (5):

(5) $$\Phi_{j}(t)=\frac{2\pi n}{60}t-(j-1)\frac{2\pi }{N}$$

From this, UCT h_j_(t) at time t is equal to the distance between the position Q of the j-th teeth and the position P of the (j−1)-th teeth at the time (t−ṷ_j_(t)), where ṷ_j_(t) is the time delay between the j-th teeth and (j−1)-th teeth. 

At time *t*, the Q point coordinate value may be expressed in Equation (6):

(6) $$\left[\begin{array}{l} Q_{x}\\ Q_{y} \end{array}\right]=\left[\begin{array}{l} O_{\mathrm{tx}}\left(t\right)\\ O_{\mathrm{ty}}\left(t\right) \end{array}\right]+\left[\begin{array}{l} x(t)\\ y(t) \end{array}\right]+\left[\begin{array}{l} R_{t}\cdot \sin(\Phi_{j}\left(t)\right)\\ R_{t}\cdot \cos(\Phi_{j}\left(t)\right) \end{array}\right]$$

In Equation (6), ${\left[\begin{array}{cc} O_{\mathrm{tx}} & O_{\mathrm{ty}} \end{array}\right]}^{T}$ is the coordinates of a moving coordinate system origin in the fixed coordinate system XOY, where ${\left[x(t\right)\;y(t)]}^{T}$ is the coordinates of the tool center in the moving coordinate system. 

At time (t−ṷ_j_(t)), the point P coordinate may be calculated from Equation (7):

(7) $$\left[\begin{array}{l} P_{x}\\ P_{y} \end{array}\right]=\left[\begin{array}{l} O_{\mathrm{tx}}(t-{\underset{\wedge }{u}}_{j}(t))\\ O_{\mathrm{tx}}(t-{\underset{\wedge }{u}}_{j}(t)) \end{array}\right]+\left[\begin{array}{l} x(t-{\underset{\wedge }{u}}_{j}(t))\\ y(t-{\underset{\wedge }{u}}_{j}(t)) \end{array}\right]+\left[\begin{array}{l} R_{t}\cdot \sin(\Phi_{j-1}(t-{\underset{\wedge }{u}}_{j}(t)))\\ R_{t}\cdot \cos(\Phi_{j-1}(t-{\underset{\wedge }{u}}_{j}(t))) \end{array}\right]$$

P point coordinate may also be calculated using Equation (8):

(8) $$\left[\begin{array}{l} P_{x}\\ P_{y} \end{array}\right]=\left[\begin{array}{l} O_{\mathrm{tx}}\left(t\right)\\ O_{\mathrm{ty}}\left(t\right) \end{array}\right]+\left[\begin{array}{l} x(t)\\ y(t) \end{array}\right]+\left[\begin{array}{l} \left(R_{t}-h_{j}\left(t)\right)\cdot \sin(\Phi_{j}\left(t\right)\right)\\ \left(R_{t}{-h}_{j}\left(t)\right)\cdot \cos(\Phi_{j}(t)\right) \end{array}\right]$$

Obviously, Equations (7) and (8) are equal, therefore,

(9) $$O_{\mathrm{tx}}{(t)=O}_{\mathrm{tx}}(t-{\underset{\wedge }{u}}_{j}(t))+\int_{{t-\tau }_{j}}^{t}v_{f}(t)dt$$

(10) $$O_{\mathrm{ty}}(t-{\underset{\wedge }{u}}_{j}(t))=O_{\mathrm{ty}}(t)$$

From Equations (7)–(10):

(11) $$\left[\begin{array}{l} x(t-{\underset{\wedge }{u}}_{j}(t))\\ y(t-{\underset{\wedge }{u}}_{j}(t)) \end{array}\right]+\left[\begin{array}{l} R_{t}\cdot \sin(\Phi_{j-1}(t-{\underset{\wedge }{u}}_{j}(t)))\\ R_{t}\cdot \cos(\Phi_{j-1}(t-{\underset{\wedge }{u}}_{j}(t))) \end{array}\right]=\left[\begin{array}{l} \int_{{t-\tau }_{j}}^{t}v_{f}(t)\mathrm{dt}\\ 0 \end{array}\right]+\left[\begin{array}{l} x(t)\\ y(t) \end{array}\right]+\left[\begin{array}{l} \left(R_{t}-h_{j}(t))\cdot \sin(\Phi_{j}(t)\right)\\ \left(R_{t}-h_{j}(t))\cdot \cos(\Phi_{j}(t)\right) \end{array}\right]$$

where

(12) $$\Phi_{j-1}(t-{\underset{\wedge }{u}}_{j}(t))=\Phi_{j}(t)-\frac{2\pi n}{60}{\underset{\wedge }{u}}_{j}(t)+\frac{2\pi }{N}$$

As a result, UCT may be calculated using Equation (13):

(13) $$\begin{array}{l} h_{j}(t)=\sin(\Phi_{j}(t))\cdot \int_{t-{\underset{\wedge }{u}}_{j}}^{t}v_{f}\left(t\right)dt+R_{t}\cdot [1-\cos\left(\frac{2\pi }{N}-\frac{2\pi n}{60}{\underset{\wedge }{u}}_{j}(t)\right)]\\ +[x(t)-x(t-{\underset{\wedge }{u}}_{j}(t))]\cdot \sin(\Phi_{j}(t))+[y(t)-y(t-{\underset{\wedge }{u}}_{j}(t))]\cdot \cos(\Phi_{j}(t)) \end{array}$$

and

(14) $$\begin{array}{ll} R_{t}\cdot \sin\left(\frac{2\pi }{N}-\frac{2\pi n}{60}{\underset{\wedge }{u}}_{j}(t)\right) & =\cos(\Phi_{j}(t))\cdot \int_{{t-\tau }_{j}}^{t}v_{f}\left(t\right)dt+(x(t)-x(t-{\underset{\wedge }{u}}_{j}(t))\cdot \cos(\Phi_{j}(t))\\ & -(y(t)-y(t-{\underset{\wedge }{u}}_{j}(t))\cdot \sin(\Phi_{j}(t)) \end{array}$$

If the tool moves uniformly with feed rate, the UCT h_j_(t) is composed of a static cutting thickness h_js_ and a dynamic cutting thickness h_jd_. Thus:

(15) $$h_{\mathrm{jd}}\left(t\right)=(x(t)-x(t-{\underset{\wedge }{u}}_{j}(t))\sin(\Phi_{j}(t))+(y(t)-y(t-{\underset{\wedge }{u}}_{j}(t))\cos(\Phi_{j}(t))$$

In actual fact, for LTUM, the cutting edge trajectory is similar to sub-cycloid motion. The cutting edge generates sub-cycloid motion with a longer period of spindle rotation and a shorter period of ultrasonic vibration. 

Thus, at time t, UCT h_jlt_(t) of LTUM is given by Equation (16):

(16) $$\begin{array}{ll} h_{\mathrm{jlt}}(t)= & f_{z}\cdot \sin[A_{t}\cdot \cos(2\pi f{t+\varphi }_{l-t})-\left(j-1\right)\cdot \frac{2\pi }{N}]{+\sin[(A}_{t}\cdot \cos(2\pi f{t+\varphi }_{l-t})-\left(j-1\right)\cdot \frac{2\pi }{N}]\\ & \cdot \left[\begin{array}{l} v_{f}\cdot {t+R}_{t}\cdot \sin\left(\omega_{n-t}\right){-v}_{f}\cdot \left(t-T\right)\\ +R_{t}\cdot \sin\left(\omega_{n-t}\left(t-T\right)\right) \end{array}\right]+\cos[(A_{t}\cdot \cos(2\pi f{t+\varphi }_{l-t})-\left(j-1\right)\cdot \frac{2\pi }{N}]\\ & \cdot \left[v_{f}\cdot {t+R}_{t}\cdot {\sin(\omega }_{n-t}{)-v}_{f}\cdot \left(t-T\right)+R_{t}\cdot {\sin(\omega }_{n-t}\left(t-T)\right)\right] \end{array}$$

### 2.3. Cutting Force Model of LTUM

**Figure 5:**
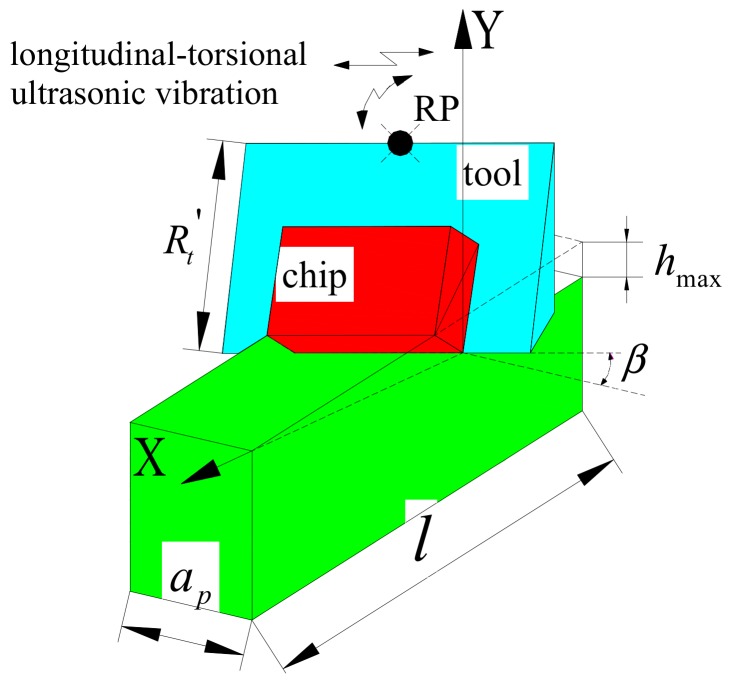
Model of oblique cutting.

**Figure 6:**
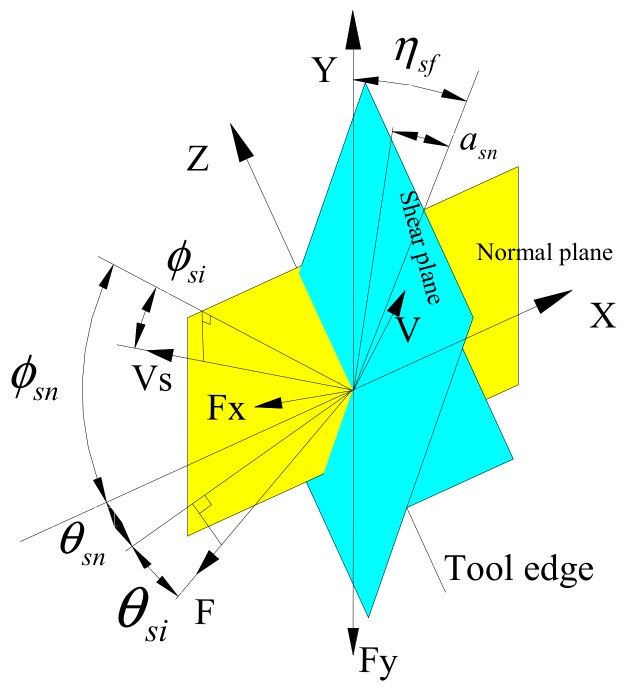
Geometric relationship of cutting force model.

On the basis of Merchant’s [23] cutting force model, we transformed LTUM into an oblique cutting model, as shown in Figure 5. The geometric relationship of this cutting force model is shown in Figure 6.

From Figure 5 and Figure 6:

(17) $$\sin\theta_{\mathrm{si}}=\sin\beta_{\mathrm{sf}}{\sin\eta }_{f}$$

(18) $${\tan(\theta }_{\mathrm{sn}}{+\alpha }_{\mathrm{sn}}{)=\tan\beta }_{\mathrm{sf}}{\cos\eta }_{f}$$

According to Ref. [24], the relationship between the chip flow direction angle and the inclination angle (helix angle) is:

(19) $$\eta_{f}=\beta$$

where β_sf_ is the friction angle and the direction of cutting speed and force is determined by ϕ_sn_, ϕ_si_, θ_sn_, θ_si_.

Then, the normal force F_n_ may be obtained using:

(20) $$F_{n}{=F}_{x}{\cos\beta \sin f}_{\mathrm{sn}}{+F}_{y}{\cos f}_{\mathrm{sn}}$$

where Fx and Fy represent the cutting forces in the feed direction and the width of cut direction, respectively. 

A non-linear cutting force model [25] is adopted. For any teeth, j, the instantaneous cutting forces are related to the UCT, as in Equation (21):

(21) $$\left\{\begin{array}{l} F_{\mathrm{tj}}{=K}_{t}a_{p}h_{jlt}{\left(t\right)}^{q}\\ F_{\mathrm{rj}}{=K}_{r}a_{p}h_{jlt}{\left(t\right)}^{q} \end{array}\right.$$

Then, Fx and Fy may be solved by applying Equation (22):

(22) $$\left[\begin{array}{c} F_{x}\\ F_{y} \end{array}\right]=\sum_{j=1}^{N}g(\Phi_{j}(t))\left[\begin{array}{cc} -\cos(\Phi_{j}\left(t)\right) & -\sin(\Phi_{j}\left(t)\right)\\ \sin(\Phi_{j}\left(t)\right) & -\cos(\Phi_{j}\left(t)\right) \end{array}\right]\left[\begin{array}{c} F_{\mathrm{tj}}\\ F_{\mathrm{rj}} \end{array}\right]$$

where $g(\Phi_{j}(t))$ is the window function. To judge whether or not the teeth are cutting in, Equation (23) is applied:

(23) $$g(\Phi_{j}(t))=\left\{\begin{array}{l} 1,\Phi_{\mathrm{st}}\leq \Phi_{j}(t)\leq \Phi_{\mathrm{ex}}\\ 0,\mathrm{otherwise} \end{array}\right.$$

where Φ_st_, Φ_ex_ is the cutting-in and out angle.

From Equations (21)–(23), cutting force at any time is solved using Equation (24):

(24) $$\begin{array}{c} F_{x}\left(t\right)\\ F_{y}\left(t\right) \end{array}=a_{p}\sum_{j=1}^{N}g(\Phi_{j}\left(t)\right)\left(\left[\begin{array}{c} {-K}_{t}\cos\Phi_{j}{(t)-K}_{r}\sin\Phi_{j}\left(t\right)\\ K_{t}\sin\Phi_{j}{(t)-K}_{r}\cos\Phi_{j}\left(t\right) \end{array}\right]{g(h}_{\mathrm{jlt}}{\left(t)\right)}^{q}\right)$$

where Kt, Kr are cutting force coefficients, calculated by Equation (25):

(25) $$\begin{array}{l} K_{t}=\frac{\tau_{\mathrm{sf}}{(\cos\theta }_{\mathrm{sn}}{+\tan\theta }_{\mathrm{si}}\tan\beta )}{\left[{\cos(\theta }_{\mathrm{sn}}+\Phi_{\mathrm{sn}})\cos\Phi_{\mathrm{si}}{+\tan\theta }_{\mathrm{si}}\sin\Phi_{\mathrm{si}}\right]\sin\Phi_{\mathrm{sn}}}\\ K_{r}=\frac{\tau_{\mathrm{sf}}{\sin\theta }_{\mathrm{sn}}}{\left[{\cos(\theta }_{\mathrm{sn}}{+\Phi }_{\mathrm{sn}}{)\cos\Phi }_{\mathrm{si}}{+\tan\theta }_{\mathrm{si}}{\sin\Phi }_{\mathrm{si}}\right]{\cos\beta \sin\Phi }_{\mathrm{sn}}} \end{array}$$

## 3. Results

A series of verification experiments were carried out under LTUM to assess the matching attributes of the established cutting force model for LTUM.

### 3.1. Experimental Condition

**Figure 7:**
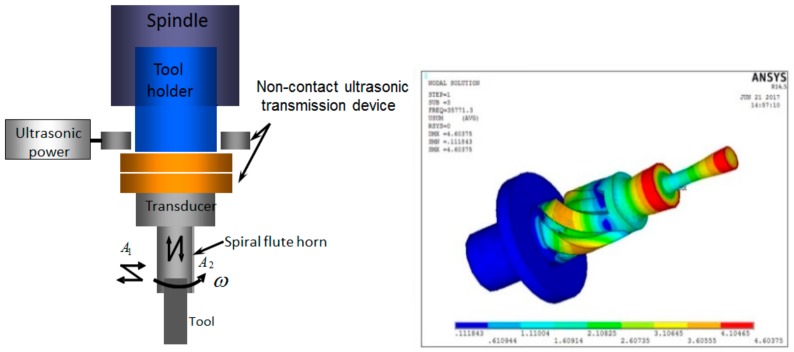
Longitudinal-torsional ultrasonic vibration system.

As shown in Figure 7, longitudinal ultrasonic vibration was transmitted from an ultrasonic power supply to a transducer via a non-contact transmission device. The longitudinal vibration was then transformed into longitudinal-torsional vibration by a horn with spiral flutes, generating longitudinal-torsional vibration on the milling tool.

**Figure 8:**
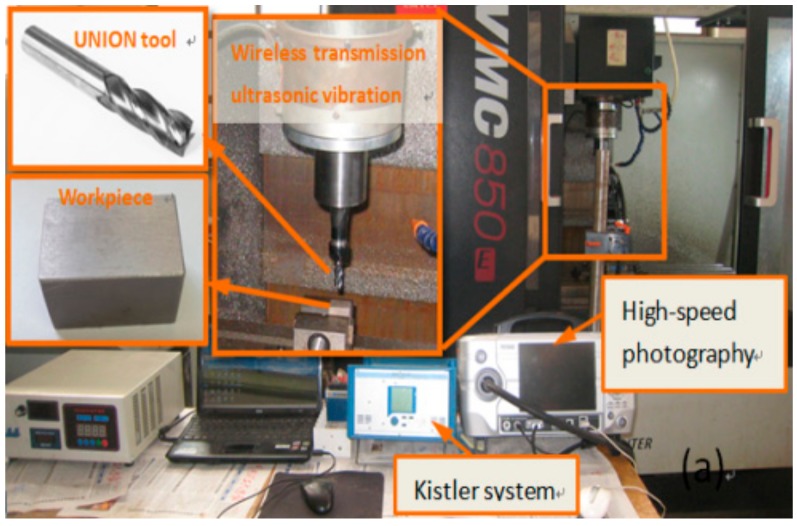
Experimental devices.

The side and down milling of Ti-6Al-4V was carried out on a machining center. The chemical composition of the workpiece material, Ti-6Al-4V was presented in Ref. [15]. The size of the rectangular workpiece used was 30 mm × 15 mm × 6 mm. The experimental equipment was composed of a Kistler dynamometer system (9257B, Winterthur, Switzerland), a self-developed wireless transmission longitudinal-torsion ultrasonic vibration assisted milling system, a high-speed photography camera and a computer. For the machining processing, a cemented carbide UNION tool (C-CES 10*25) was adopted. The experimental devices are shown in Figure 8.

### 3.2. Experimental Verification Results 

In order to verify the established cutting force model, the calculated cutting force result was compared with the experimental result. This was carried out based on the cutting force direction exhibited in Figure 1.

**Figure 9:**
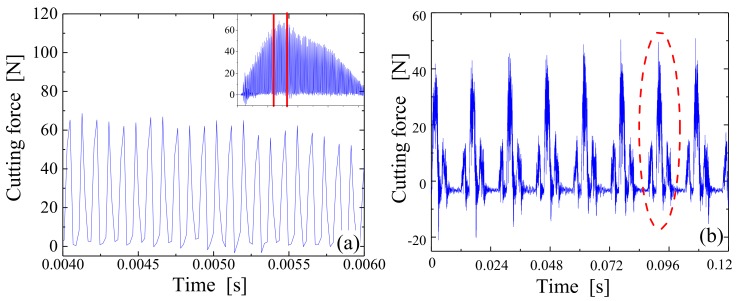
Predicted and experimental results of cutting force (cutting speed is 25 m/min, feed per tooth is 0.008 mm/z, width of cut is 0.15 mm, rake angle is 5°, relief angle is 12°, helix angle is 30°, cutting edge radius is 0.01 mm, longitudinal amplitude is 5 μm, torsional amplitude is 4 μm, and frequency is 35 kHz). (**a**) Model simulated result; (**b**) Experimental result.

The simulated result is shown in Figure 9a. The cutting force appeared to first increase and then decrease after reaching a peak value, resulting in a parabolic trend considered in accordance with a variation law of undeformed chip thickness. The cutting force was also found to periodically return to zero with the ultrasonic vibration, which was mainly caused by the tool–workpiece separation characteristics produced in LTUM.

The experimental result is shown in Figure 9b. Again, the cutting force was found to fluctuate periodically with the cutting-in and cutting-out of teeth; however, the result was different from the simulated findings as the cutting force did not return to zero. The reason for this is it is impossible to accurately measure the cutting force of each ultrasonic cycle, even when using a Kistler 9257B piezoelectric dynamometer (Winterthur, Switzerland), which is one of the most-advanced dynamometers in the world (with a sampling frequency of 10 kHz).

**Figure 10:**
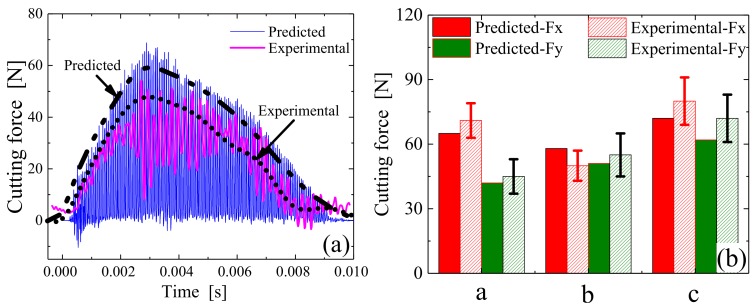
Cutting force comparison of predicted and experimental results. (**a**) Under single tooth; (**b**) Under different processing parameters.

The simulated and experimental results were further compared, as shown in Figure 10. Figure 10a is a comparison of the cutting force for individual teeth (e.g., the area circled in red in Figure 9b), with the same processing parameters outlined in Figure 9. Figure 10b compares the model-predicted and experimental cutting force results under different parameters, wherein CS is the abbreviation for cutting speed, FpT is the feed per tooth, WoC is the width of cut, DoC is the depth of cut, LA is the longitudinal amplitude, and TA is the torsional amplitude. The relevant processing parameters for this comparison are provided in Table 1, Table 2 and Table 3. Although it was not possible to compare the cutting force of each ultrasonic cycle, the predicted result appeared to be in good agreement with the experimental result based on the whole view, suggesting that the established cutting force model worked well in such conditions.

**Figure 11:**
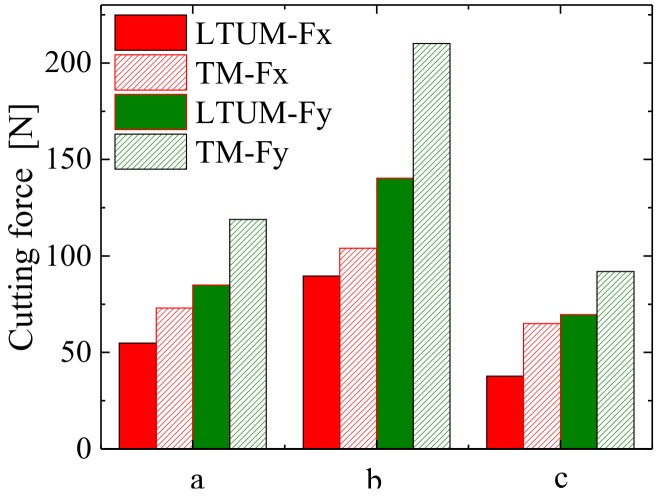
Cutting force comparison of LTUM and TM.

Figure 11 shows a comparison of the cutting forces in TM and LTUM, wherein the processing parameters are listed in Table 1, Table 2 and Table 3 (for TM, the Table 3 data are set to 0). Compared with TM, the cutting forces were found to be lower in LTUM, with a 24.8% decrease in Fx and a 29.9% decrease in Fy. The reason for these results is the tool and workpiece separate periodically in LTUM due to ultrasonic vibration, causing the actual cutting time of LTUM to be less than that of TM. In addition, with the longitudinal and torsional ultrasonic vibration, the friction resistance between the chip and the tool is transformed into a beneficial cutting force, so that the cutting force is smaller than that of TM.

## 4. Discussion

### 4.1. Influence of Tool Geometry Parameters on Cutting Force

The geometric parameters of tools have a great influence on the cutting force and machining quality. However, it is difficult to evaluate the influence of tool geometry parameters on machining results by experimental methods. On the one hand, the cost is high and the efficiency is low; on the other hand, the choice of geometric parameters of the formed tool (especially a vertical milling tool) is limited, and the time and cost involved in tool parameter customization is high. By using theoretical simulations, the influence of tool geometry parameters on machining results can be analyzed economically and efficiently, providing another option for engineering applications.

**Figure 12:**
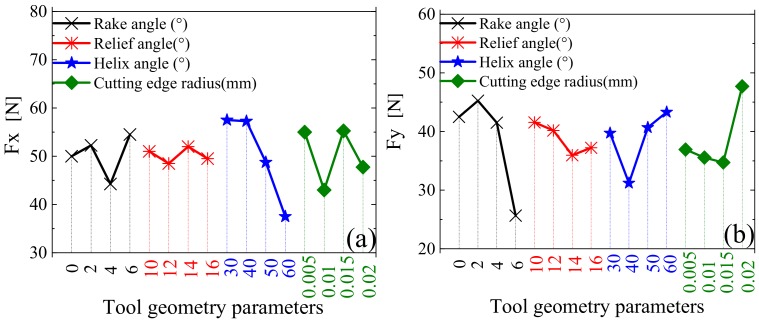
Response relationships between tool geometry parameters and cutting force. (**a**) Cutting force Fx; (**b**) Cutting force Fy.

Using an orthogonal design of a model simulation for tool parameters, which is presented in Table 4 along with other relevant parameters listed in Table 5, the influence of tool geometry parameters on cutting force was calculated. The results are shown in Figure 12, where Fx represents the feed direction and Fy represents the width of cut direction.

In Figure 12, it can be seen that, within the range of the selected parameters: Fx fluctuates and Fy decreases with an increase in the rake angle; both Fx and Fy exhibit a small decrease with an increase in the relief angle; Fx decreases and Fz noticeably increases with increasing helix angle; and, with an increase in the cutting edge radius, Fx is stable and Fy increases.

**Figure 13:**
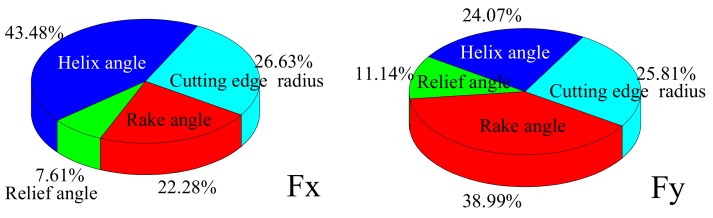
Relative sensitivity of tool geometry parameters to cutting force.

The sensitivity of tool geometry parameters to the cutting force is shown in Figure 13. As can be seen, for Fx, helix angle had the greatest influence, cutting edge radius and rake angle had the second largest effects, and relief angle had the least influence. For Fy, the rake angle had the greatest influence, followed closely by the cutting edge radius and helix angle, while the relief angle had the least influence. Thus, it is obvious that the tool geometry parameters had different influences on the cutting forces.

### 4.2. Influence of Ultrasonic Parameters on Cutting Force

In LTUM, the influence of ultrasonic parameters (longitudinal amplitude, torsional amplitude, frequency and longitudinal-torsional phase difference) on the processing results cannot be neglected. However, in the experiment, it was very difficult to obtain a series of ultrasonic parameters by changing the acoustic system. Accordingly, our theoretical model realized the analysis of the influence of ultrasonic parameters on the processing results both economically and efficiently.

**Figure 14:**
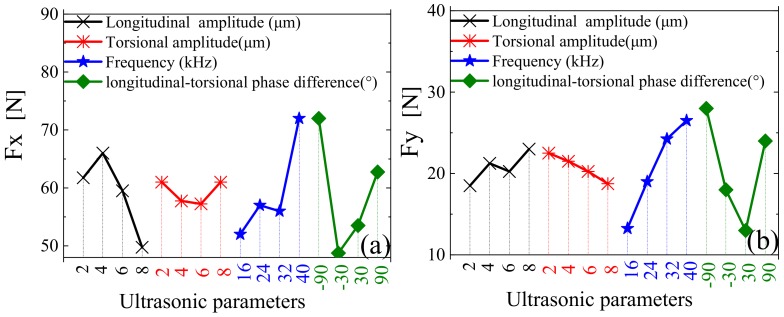
Response relationships between ultrasonic parameters and cutting force. (**a**) Cutting force Fx; (**b**) Cutting force Fy.

According to the orthogonal design, four factors and four levels were adopted, as shown in Table 6. The simulation results are shown in Figure 14, while the cutting and tool geometry parameters are shown in Table 7.

In Figure 14, it can be seen that, within the range of the selected parameters: Fx decreases and Fy fluctuates over a small range with an increase in longitudinal amplitude; Fx fluctuates over a small range and Fy decreases with increasing torsional amplitude; Fx and Fy increase with increasing ultrasonic frequency; and, with increasing longitudinal-torsional phase difference, Fx and Fy first decrease and then increase, reaching a minimum value at 30°. 

**Figure 15:**
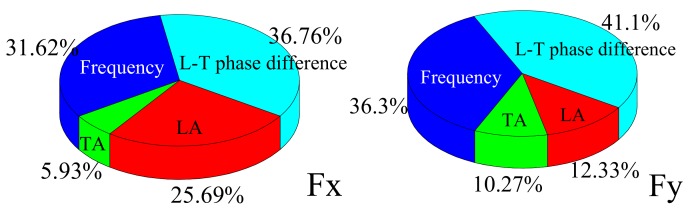
Relative sensitivity of ultrasonic parameters to cutting force.

The sensitivity of ultrasonic parameters to cutting force is shown in Figure 15. As can be seen, for both Fx and Fy, longitudinal-torsional phase difference had the greatest influence, followed closely by ultrasonic frequency and longitudinal amplitude, while torsional amplitude had the least influence. 

### 4.3. Influence of Milling Parameters on Cutting Force

**Figure 16:**
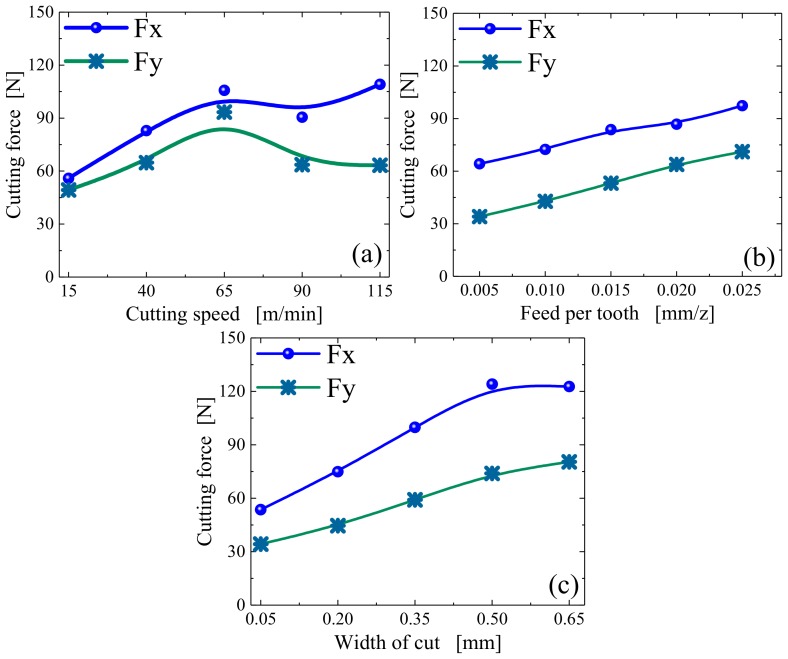
Response relationships between cutting force and milling parameters. (**a**) Under different cutting speed; (**b**) Under different feed per tooth; (**c**) Under different width of cut.

In addition to tool geometry and ultrasonic parameters, milling parameters also have a great influence on cutting force. According to the orthogonal design, four factors and four levels were adopted, as shown in Table 8, with the simulation results shown in Figure 16.

As can be seen in Figure 16a, with an increase in the cutting speed, both Fx and Fy gradually increase and then exhibit a small decrease in the final stage. The main reasons for this phenomenon are as follows: Firstly, with increasing cutting speed, the material removal volume of per unit time increases, resulting in a gradual increase in cutting force. Secondly, the critical cutting speed has a greater influence on the separation characteristics in LTUM; thus, with an increase in cutting speed, the phenomenon of tool–chip separation is weakened, causing the cutting force to increase gradually. In addition, an increase in cutting speed increases the material removal rate, causing a gradual increase in the cutting heat, thereby increasing of the workpiece temperature. This has an influence on the physical properties of the materials, such that the cutting force has a certain degree of decline when the cutting speed reaches a specific value.

From Figure 16b,c, with increasing of feed per tooth and width of cut, the cutting forces Fx and Fy increase in varying degrees, and the trend is similar. The main reason is that with increasing of feed per tooth and width of cut, the material removal per unit time increases as well, caused shear force increases, resulting in the gradual increase of cutting force.

**Figure 17:**
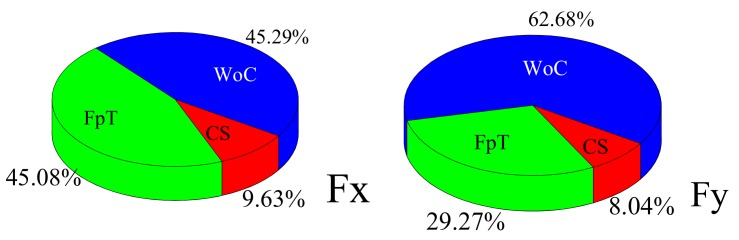
Relative sensitivity of milling parameters to cutting force.

The sensitivity of ultrasonic parameters to cutting force is shown in Figure 17. As can be seen, the width of cut had the greatest influence on Fx and Fy, with a contribution rate of 45.29% and 62.68%, respectively. The feed per tooth followed closely, with a contribution rate of 45.08% and 29.27% for Fx and Fy, respectively. The cutting speed had the least influence for both cutting force directions. The main reason for this is that cutting force has a direct relationship with undeformed chip thickness, while both width of cut and feed per tooth have a great influence on undeformed chip thickness. Therefore, width of cut and feed per tooth have greater influences on the cutting force.

## 5. Conclusions

In this work, an LTUM machining method was proposed to meet the compressive stress and anti-fatigue manufacturing requirements of titanium alloy Ti-6Al-4V. In addition, models of cutting edge trajectory and undeformed chip thickness were presented. From these, a cutting force model of the LTUM method was established and verified through a series of experiments. Based on the established model, the influence of several parameters on cutting force were able to be analyzed. The major conclusions of this work are:

(1) The undeformed chip thickness of the LTUM method was quite different from TM, and had a great influence on cutting force.
(2) Based on the experimental verification, the established theoretical model of cutting force was in good agreement with the experimental results.
(3) Based on the established cutting force model, the cutting force was lower in LTUM compared to TM. Specifically, Fx decreased by 24.8% and Fy decreased by 29.9%.
(4) Based on the established cutting force model, the tool geometry parameters, ultrasonic parameters and milling parameters each influenced the cutting forces differently.

## Figures and Tables

**Table 1:** Milling parameters.

Group	CS (m/min)	FpT (mm/z)	WoC (mm)	DoC (mm)
(a)	25	0.008	0.15	2
(b) and (c)	40	0.005	0.1	2

**Table 2:** Tool geometry parameters.

Group	Rake Angle (°)	Relief Angle (°)	Helix Angle (°)	Cutting Edge Radius (mm)
(a), (b) and (c)	5	12	30	0.01

**Table 3:** Ultrasonic parameters.

Group	LA (μm)	TA (μm)	Frequency (kHz)
(c)	5	4	35
(a) and (b)	4	3.2	

**Table 4:** Simulated design for tool geometry parameters L_16_ (4^5^).

Lever	Rake Angle (°)	Relief Angle (°)	Helix Angle (°)	Cutting Edge Radius (mm)
No. 1	0	10	30	0.005
No. 2	2	12	40	0.01
No. 3	4	14	50	0.015
No. 4	6	16	60	0.02

**Table 5:** Cutting and ultrasonic parameters.

CS (m/min)	FpT (mm/z)	WoC (mm)	DoC (mm)	LA (μm)	TA (μm)	Frequency (kHz)
25	0.008	0.15	2	2	2	20

**Table 6:** Design for ultrasonic parameters L_16_ (4^5^).

Lever	LA (μm)	T (μm)	Frequency (kHz)	Phase Difference (°)
No. 1	2	2	16	−90
No. 2	4	4	24	−30
No. 3	6	6	32	30
No. 4	8	8	40	90

**Table 7:** Cutting and tool geometry parameters.

CS (m/min)	FpT (mm/z)	WoC (mm)	DoC (mm)	Rake Angle (°)	Relief Angle (°)	Helix Angle (°)	Cutting Edge Radius (μm)
30	0.006	0.12	2	6	10	25	5

**Table 8:** Design for milling parameters L_16_ (4^5^).

Lever	CS (m/min)	FpT (mm/tooth)	WoC (mm)
No. 1	20	0.006	0.1
No. 2	40	0.012	0.2
No. 3	60	0.018	0.3
No. 4	80	0.024	0.4
